# Dietary ethylenediamine dihydroiodide improves intestinal health in Cherry Valley ducks

**DOI:** 10.1016/j.psj.2023.103022

**Published:** 2023-08-11

**Authors:** Yueqin Xie, Jing Li, Dongyun Liu, Bing Wu, Hua Zhao, Guangmang Liu, Gang Tian, Jingyi Cai, Caimei Wu, Jiayong Tang, Gang Jia

**Affiliations:** ⁎ Institute of Animal Nutrition, Key Laboratory for Animal Disease-Resistance Nutrition of China, Ministry of Education, Sichuan Agricultural University, Chengdu, Sichuan, 611130, China; † Sichuan Jilongda Co., Ltd, Mianyang, Sichuan, 618000, China

**Keywords:** ethylenediamine dihydroiodine, thyroid function, intestinal barrier, gut microbiota, intestinal health

## Abstract

This study investigated the effect of ethylenediamine dihydroiodide (**EDDI**) on the growth performance, thyroid function, immune function, intestinal development, intestinal permeability, intestinal barrier functions and microbial characteristics of Cherry Valley ducks. The results showed that the addition of EDDI significantly increased body weight, average daily gain, serum level of lymphocytes, basophils, triiodothyronine, thyroxine and thyrotropin, villus height, and villus height-to-crypt depth ratio, and significantly decreased crypt depth, diamine oxidase, serum D-Lactic acid of ducks (*P* < 0.05). EDDI also significantly up-regulated the mRNA expression of zonula occludens-1, zonula occludens-2, zonula occludens-3, mucin 2, secretory immunoglobulin A, interleukin-10 and avian β-defensin 2 in the jejunum and ileum (*P* < 0.05), and down-regulated the mRNA expression of occludin and interleukin-6 in the jejunum and ileum. Additionally, the addition of EDDI significantly increased cecal level of acetic acid, propionic acid, butyric acid (*P* < 0.05). Cecal microbiome analysis indicated that the addition of EDDI significantly increased the relative abundance of these microorganisms that can produce short-chain fatty acids, mainly including *Actinobacteria, Verrucomicrobia, Clostridiales* and *Lactobacillales*, and decreased the relative abundance of pathogenic bacteria *Deferribactere*. Interestingly, triiodothyronine and thyroxine levels were highly positively correlated with the relative abundance of *Actinobacteria*. These results revealed that the addition of EDDI could promote the growth and development of meat ducks by improving their thyroid function, immune function, intestinal development and intestinal barrier functions of ducks.

## INTRODUCTION

Iodine is one of the essential trace elements in animals, which promotes the growth performance of animals by regulating metabolism, enhancing immunity, eliminating reactive oxygen species, and other physiological functions (Iannaccone et al., 2019). Sirakov and Plateroti (2011) found that iodine deficiency or excess caused thyroid dysfunction, led to decreased immune function, and then affect the growth and development of the body. At present, the dietary iodine proposed by the National Research Council may not meet the nutritional requirements of fast-growing poultry, and its genetic growth potential ca not be fully exerted (Inal et al., 2001). Therefore, in the current poultry breeding process, iodine is mainly added to trace mineral premix in inorganic form (Ammerman and Miller, 1972). However, a study reported that inorganic trace minerals have high loss rate and low utilization rate, but organic chelated trace minerals can solve these problems to some extent because of their higher bioavailability (Bao and Choct, 2009; Yu et al., 2022).

Additionally, the growth and development of poultry is also closely related to intestinal health. It has been reported that the intestinal barrier dysfunction is considered as one of the main reasons for the decrease of production performance and the increase of health problems in poultry (Lambert, 2009). However, in the current intensive farming process, factors such as high-density farming, heat stress and toxins will cause intestinal barrier dysfunction of poultry, destroy intestinal health, lead to reduced production performance, and thus cause serious economic losses (Goo et al., 2019). Therefore, it is still one of the important challenges in the field of poultry nutrition research to increase the growth performance by improving the intestinal barrier function and maintaining the intestinal health of ducks through nutritional strategies.

Fomichev et al. (2019) found that organic iodine can regulated the microbial composition of sheep to maintain intestinal health. Additionally, organic iodine also significantly improved the antibacterial activity and bioavailability of broilers (Ledesma et al., 2018). The result of the earlier studies indicated that organic iodine has a positive effect on the intestinal health of animals. Ethylenediamine dihydroiodide (**EDDI**) is an easily available organic chelated iodine (Ammerman et al., 1995). However, there was no scientific research on the positive effects of EDDI on the intestinal health of animals. In view of this fact, we hypothesized that EDDI could improve the intestinal health and growth performance of Cherry Valley ducks by positively influencing their thyroid function, immune function, intestinal development, intestinal barrier function and microbial characteristics.

## MATERIALS AND METHODS

### Animals and Experimental Design

A total of 360 one-day-old Cherry Valley ducks (Sichuan Mianying Duck Industry Co., Ltd., Mianyang, China) were randomly divided into 6 groups with 6 replicates in each group and 10 ducks in each replicate. The ducks fed diets with 0, 1, 2, 4, 8, and 16 mg/kg iodine (EDDI form, Jilongda Co., Ltd, Guanghan, China) for 35 d. The nutritional components and requirements were listed in Table S1. The iodine concentration of the 6 diets were analyzed to be 0.82, 1.90, 3.23, 4.35, 9.27, 17.16 mg/kg for the diets at the age of 1 to 14 d, and 0.97, 2.05, 3.54, 5.47, 9.86, 17.65 mg/kg for the diets at the age of 15 to 35 d. The weight and feed consumption of ducks were recorded at each stage during the experiment.

### Sample Collection

Ducks were fasted for 12 h on 35 d, and an average weight duck (n = 6) was randomly selected from each repetition. After 10 mL of blood was collected by jugular vein and centrifuged at 4°C for 3,500 r/min for 15 min to separate serum, which was placed in a refrigerator at -20°C for analysis. The selected experimental ducks (36 ducks) were anesthetized and slaughtered. The jejunum and ileum segments with a length of 4 cm were washed with PBS and fixed in 4% paraformaldehyde for histomorphological analysis. Additionally, jejunum and ileum tissues were collected and frozen in liquid nitrogen, and then stored to -80°C for indicator analysis. The contents of caeca were removed and collected in sterile 1.5-mL tubes. Then caecum contents were flash-frozen in liquid N_2_ and stored at –80°C until microbial community analysis.

### Hematological Analyses

Hematological index including lymphocytes, basophils and eosinophils, were detected by automatic analyzer (Coulter Max M, Coulter Electronics Ltd, Luton, UK). Additionally, the levels of triiodothyronine (**T3**), thyroxine (**T4**) and thyrotropin (**TSH**) were examined by kit (Chengdu Weskong Bioscience and Technology Co., Ltd, Chengdu, China), and the specific steps were carried out according to the instructions.

### Histomorphological Analysis

After dehydration, pruning, embedding, slicing and hematoxylin-eosin staining, the intestinal segment was observed by BA210Digital microscope camera (Motic, Xiamen, China), and the areas with better slices were randomly selected for photographing, and the villi height (**VH**) and crypt depth (**CD**) of each slice were measured, and the ratio of villi to crypt (**V: C**) was calculated on the basis of the method reported by Qin et al. (2019).

### Determination of Diamine Oxidase and d-Lactate

The levels of diamine oxidase (**DO**) and D-lactic acid (**D-LA**) in serum were determined by ELISA kit. The kit was purchased from Chengdu Micro-Space Biotechnology Co., Ltd. (Chengdu, China), and the results were read by SpectraMax 190 full-wavelength microplate reader.

### Quantitative Real-Time PCR

Total RNA was isolated from frozen jejunum and ileum tissue using trizol reagent (Takara, Beijing, China) according to the manufacturer's specifications. Real-time quantitative PCR was conducted using the primers listed in Table S2. All PCR contained 5.0 μL of SYBR Green qPCR Mix, 0.2 μL of cDNA, 0.3 μL of each primer, and 4.2 μL of double distilled water in a final volume of 20 μL. Amplification program was 95°C 15 min, followed by forty cycles of 95°C 5 s and 60°C 30 s, and a final melting curve analysis. The reference gene β-actin served as a control to normalize the mRNA expression level. Intestinal barrier-related genes mainly including physical barrier (zonula occludens-1 [***ZO-*1**], zonula occludens-2 [***ZO-*2**], zonula occludens-3 [***ZO-*3**], and occludin [***OCLN***]), chemical barrier (mucin 2 [***MUC*2**], trefoil factor 2 [***TFF2***]), immune barrier (interleukin-6 [***IL-*6**], interleukin-10 [***IL-*10**], interleukin-22 [***IL-*22**], avian β-defensin 2 [***AvBD-2***], secretory immunoglobulin A [***sIgA***] and tumor necrosis factor a [***TNF-a***]).

### Determination of Short Chain Fatty Acids

Approximately 0.5g cecum contents were mixed in 2 mL ultrapure water and centrifuged at 3,000 *g* for 15 min. The supernatant was mixed with ice-cold metaphosphoric acid solution at 4°C for 30min. After centrifugation again, the concentrations of acetic acid (**AA**), propionic acid (**PA**), isobutyric acid (**IBA**), butyric acid (**BA**), valeric acid (**VA**), and isovaleric acid (**IVA**) were determined by GC system (Varian, GC CP3800).

### Microbiome Analysis

16S rDNA gene sequencing analysis was performed by shanghai applied protein technology Co., Ltd. (China, Shanghai). The total genome DNA of the cecal contents was extracted using CTAB/SDS method. After detecting the integrity of DNA, the concentration of extracted DNA samples were measured using a NanoDrop Spectrophotometer, and only qualified samples were used for 16S rDNA sequencing analysis. The 16S rDNA V3-V4 region (Forward primer: CCTACGGGRBGCASCAG; Reverse primer: GGACTACNNGGGTATCTAAT) was selected for PCR amplification. After the PCR amplification products were purified, sequencing was performed using the Illumina Miseq sequencing platform (NovaSeq PE250). After the raw reads were spliced, filtered and de-chimeric, the last available data (high-quality clean tags) was subjected to operational taxonomic units (**OTUs**) clustering analysis according to the principle of 97% similarity. The species classification information corresponding to each OTU could be obtained through annotation in the SILVA database through OTU sequence alignment (Liu et al., 2021).

### Statistical Analyses

The experimental data were preliminarily sorted and calculated by Microsoft office 2016. SPSS 21.0 (SPSS Inc., Chicago, IL) software was used for 1-way analysis of variance among different levels of EDDI treatment groups, and Duncan method was used for multiple comparisons. Quadratic curve model fitting was used to determine the appropriate dosage of iodine added in the form of EDDI in duck diet. The experimental results were presented by the mean ± standard error, and *P* < 0.05 was regarded as significant difference. Pearson correlation coefficient between cecal microbial abundance and phenotypic characteristics was analyzed by SPSS 21.0.

## RESULTS

### Growth Performance Response to Dietary EDDI Supplementation

As shown in Table 1, the addition of 2, 4, 8, and 16 mg/kg EDDI significantly increased (*P* < 0.05) the BW of ducks at the d 14 and d 35 compared with the control group. The addition of 4 and 8 mg/kg EDDI significantly increased (*P* < 0.05) the ADG of ducks at the d 1 to d 14. Additionally, the ADG of ducks at the d 1 to d 35 was also significantly increased (*P* < 0.05) in the 4 mg/kg EDDI group. However, no differences in ADG of the ADG of ducks at the specified d 15 to d 35, as well as the ADFI and F/G in all periods (*P* > 0.05). Interestingly, the ADG of ducks at the d 1 to d 35 and in the BW of ducks at the d 14 and d 35 showed linear and quadratic relationship with EDDI (*P* < 0.05). Based on the quadratic regression analysis for BW of ducks at the d 14, d 35 and the ADG of ducks at the d 1 to d 35, the optimal dietary EDDI of ducks was estimated to be 7.40, 9.60, and 9.40 mg/kg (as iodine), respectively.

**Table 1:** Effect of different levels of EDDI on growth performance in Cherry Valley ducks.

Items	EDDI (mg/kg)						SEM	*P*-value		
	0	1	2	4	8	16		ANOVA	Linear	Quadratic
BW, g										
d 1	55.98	55.83	56.00	55.88	55.85	55.85	0.04	0.718	0.001	0.027
d 14	757.47^c^	779.37^ab^	779.41^ab^	789.44^a^	793.57^a^	783.17^a^	25.74	0.016	0.028	0.005
d 35	2453.13^c^	2496.83^bc^	2559.75^ab^	2654.17^a^	2605.83^ab^	2584.67^ab^	0.02	0.027	0.012	0.009
ADG, g										
d 1 to d 14	50.10^b^	51.68^ab^	51.67^ab^	52.40^a^	52.69^a^	49.80^b^	0.28	0.030	0.490	0.055
d 15 to d 35	80.75	81.79	84.78	88.80	86.29	87.21	0.90	0.081	0.177	0.221
d 1 to d 35	68.49^b^	69.74^b^	71.54^ab^	74.24^a^	72.85^ab^	71.54^ab^	0.57	0.022	0.001	0.002
ADFI, g										
d 1 to d 14	75.99	78.24	78.66	79.08	81.25	77.53	0.90	0.709	0.360	0.354
d 15 to d 35	176.09	170.69	173.83	182.40	182.36	182.80	1.53	0.103	0.016	0.052
d 1 to d 35	136.05	133.71	135.76	141.07	141.92	140.69	1.17	0.207	0.025	0.084
F/G, g/g										
d 1 to d 14	1.51	1.52	1.52	1.51	1.54	1.56	0.02	0.941	0.350	0.571
d 15 to d 35	2.18	2.09	2.06	2.06	2.15	2.10	0.02	0.177	0.498	0.120
d 1 to d 35	1.99	1.92	1.90	1.90	1.97	1.95	0.01	0.265	0.870	0.140

Parameters	Regression equation, mg/kg				Maximum				*P*	*R*^2^

BW d 14	*Y* = −0.5843 *χ*^2^ + 8.6457 *χ* + 763.94				2.9263				0.030	7.40
BW d 35	*Y* = −1.9998 *χ*^2^ + 38.384 *χ* + 2474.4				2.7536				0.009	9.60
ADG d 1 to d 35	*Y* = −0.0622 *χ*^2^ + 1.1358 *χ* + 69.067				2.7604				0.022	9.40

Abbreviations: ADFI, average daily feed intake; ADG, average daily gain; BW, body weight; F/G, feed-to-gain ratio.

a-c Mean values (n = 18) in a row without a common superscript are significantly different in variance analysis (*P* ˂ 0.05).

### Immune Function and Thyroid Function Response to Dietary EDDI Supplementation

The impact of EDDI on immune and thyroid function of ducks was presented in Table 2. Compared with the control group, the levels of lymphocytes, T3 and T4 in the blood of ducks in the addition of EDDI group were significantly increased (*P* < 0.05). And the addition of 16 mg/kg EDDI also significantly increased (*P* < 0.05) the level of basophils compared with the control group. However, the levels of eosinophils and TSH was not found to be significantly different among the experimental groups (*P* > 0.05).

**Table 2:** Effect of different levels of EDDI on routine hematological and thyroid function parameters in Cherry Valley ducks.

Items	EDDI (mg/kg)						SEM	*P*-value		
	0	1	2	4	8	16		ANOVA	Linear	Quadratic
Lymphocytes, %	62.2^c^	74.90^b^	81.50^ab^	83.42^ab^	85.25^ab^	92.53^a^	2.44	0.001	<0.001	<0.001
Basophils, 10^9^/L	0.20^b^	0.18^b^	0.11^b^	0.16^b^	0.08^b^	0.45^a^	0.03	0.009	0.271	0.033
Eosinophils, 10^9^/L	0.75	0.33	0.44	0.44	0.32	0.23	0.07	0.501	0.100	0.240
T_3_, pmol/L	29.25^c^	31.85^b^	34.80^a^	34.98^a^	35.56^a^	36.44^a^	0.53	<0.001	0.002	0.004
T_4_, pmol/L	678.19^c^	713.06^b^	735.50^ab^	748.36^ab^	763.28^ab^	786.47^a^	9.05	0.004	<0.001	<0.001
TSH, mIU/L	19.72	15.76	17.83	19.05	19.63	23.21	0.47	0.141	0.111	0.048

Abbreviations: T_3_, triiodothyronine; T_4_, thyroxine; TSH, thyroid-stimulating hormone.

a-c Mean values (n *=* 6) in a row without a common superscript are significantly different in variance analysis (*P* ˂ 0.05).

### Integrity of Intestinal Mucosal Morphology and Intestinal Permeability in Response to Dietary EDDI Supplementation

**Figure 1:**
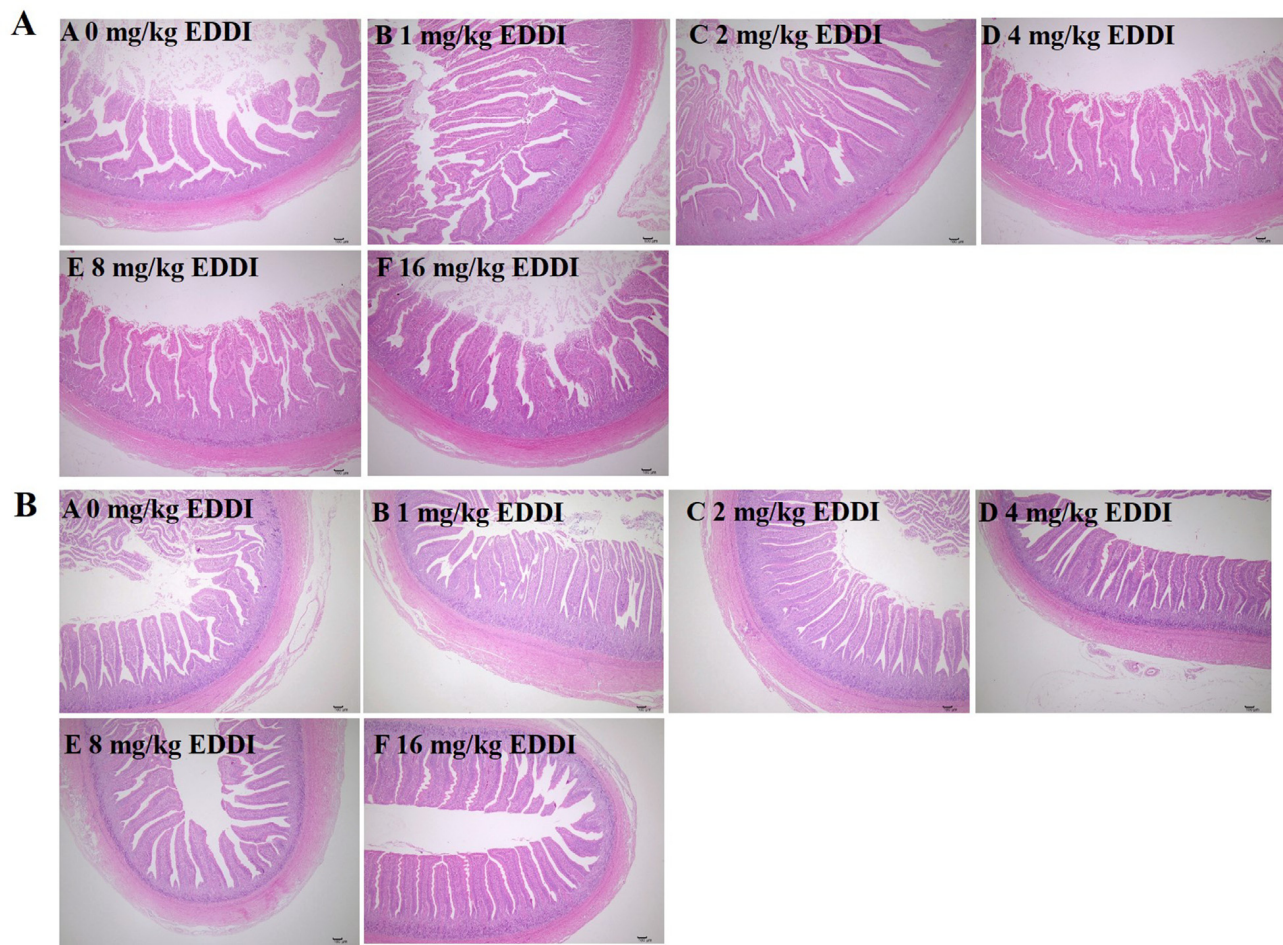
Effect of dietary EDDI on the histological structure of the jejunum and ileum in Cherry Valley ducks (magnification 10×, scale bar = 100 µm). (A) The histological structure of the jejunum in each group. (B) The histological structure of the ileum in each group. Capital letters A–F refer to 0, 1, 2, 4, 8, and 16 mg/kg EDDI groups, respectively.

Table 3 and Figure 1 showed the integrity of intestinal mucosal morphology. Compared with the control group, the VH of ileum was significantly improved in all EDDI addition groups (*P* < 0.05). Additionally, the 16 mg/kg EDDI group significantly increased the V/C of jejunum, decreased the CD of ileum (*P* < 0.05) compared with the control group. Table 4 showed the effect of EDDI on intestinal permeability of ducks, compared with the control group, the addition of 4, 8 and 16 mg/kg EDDI significantly decreased the content of DAO and D-LA of ducks (*P* < 0.05).

**Table 3:** Effect of different levels of EDDI on intestinal morphology in Cherry Valley ducks.

Items	EDDI (mg/kg)						SEM	*P*-value		
	0	1	2	4	8	16		ANOVA	Linear	Quadratic
Jejunum										
VH, μm	842.43^b^	834.96^b^	820.72^b^	790.33^b^	821.19^b^	903.45^a^	13.74	0.025	0.005	0.008
CD, μm	138.63	146.89	141.67	122.36	141.17	129.16	5.75	0.443	0.240	0.504
V/C	4.87^c^	5.68^b^	5.79^ab^	6.42^ab^	5.81^ab^	6.91^a^	0.27	0.125	0.163	0.329
Ileum										
VH, μm	664.00^c^	757.96^b^	833.41^a^	817.81^ab^	791.68^ab^	761.79^b^	16.39	<0.001	0.265	<0.001
CD, μm	122.18^b^	133.41^b^	143.46^ab^	155.04^a^	133.31^b^	100.29^c^	6.20	0.002	0.066	<0.001
V/C	6.48	6.38	5.93	5.31	6.28	7.52	0.27	0.595	0.719	0.242

Abbreviations: CD, crypt depth; V:C, villus height-to-crypt depth ratio; VH, villus height.

a-c Mean values (n = 6) in a row without a common superscript are significantly different in variance analysis (*P* ˂ 0.05).

**Table 4:** Effect of different levels of EDDI on the intestinal permeability in Cherry Valley ducks.

Items	EDDI (mg/kg)						SEM	*P*-value		
	0	1	2	4	8	16		ANOVA	Linear	Quadratic
DO, U/Ml	19.3^a^	18.04^ab^	16.13^b^	14.29^bc^	13.54^c^	14.32^b^	0.29	<0.001	<0.001	<0.001
D-LA, μmol/L	37.35^a^	35.34^ab^	34.58^ab^	32.86^b^	32.05^b^	30.05^c^	0.43	0.026	0.044	0.040

Abbreviations: D-LA, D-Lactic acid; DO, diamine oxidase.

a-c Mean values (n = 6) in a row without a common superscript are significantly different in variance analysis (*P* ˂ 0.05).

### Intestinal Barrier Function Response to Dietary EDDI Supplementation

**Figure 2:**
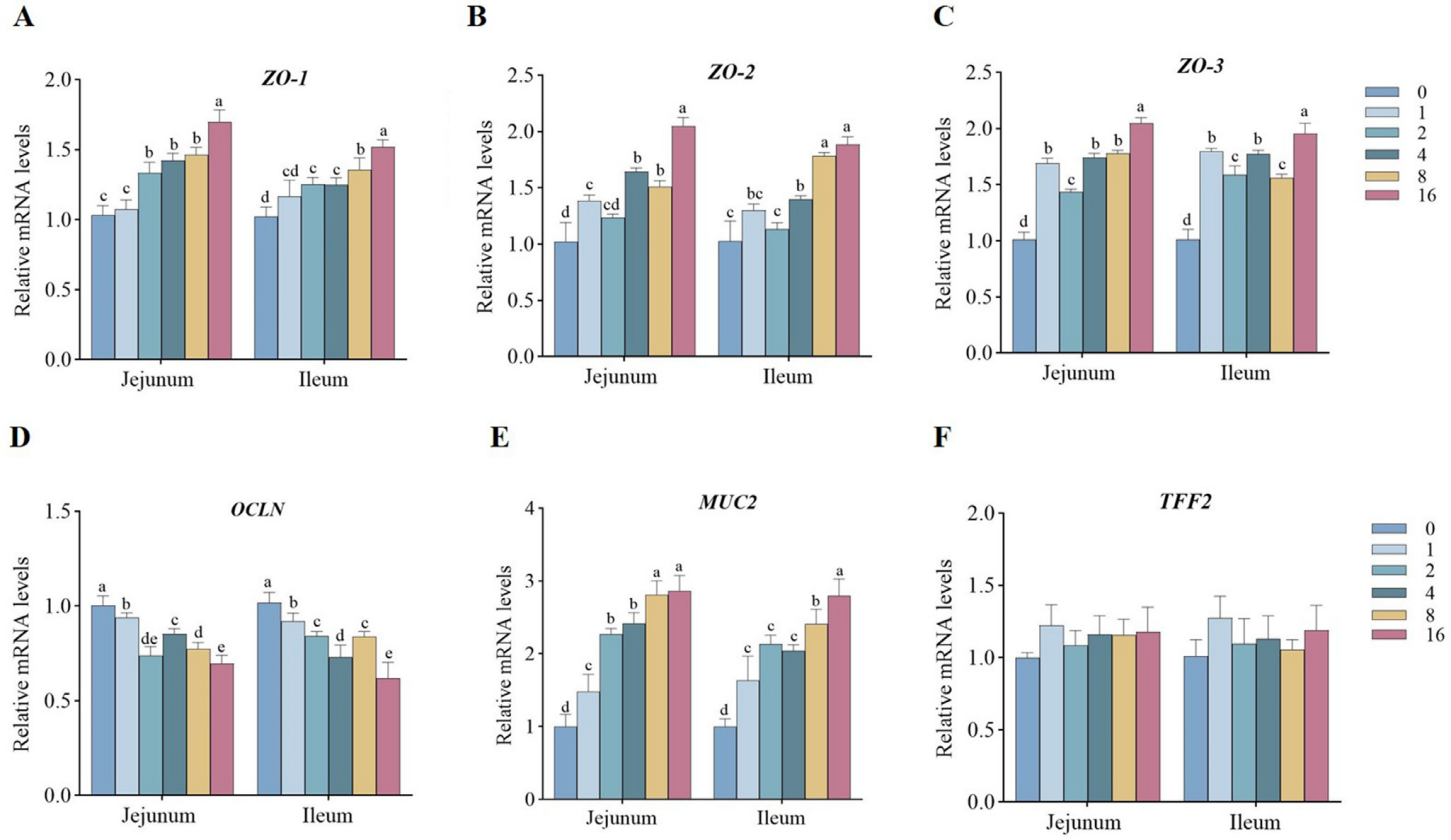
Expression of mRNA levels involved in intestinal physical and chemical barrier function of ducks fed differing levels of EDDI diets. Abbreviations: MUC2, mucin2; OCLN, occluding; TFF2, trefoil factor 2; ZO-1, zonula occludens 1; ZO-2, zonula occludens 2; ZO-3, zonula occludens 3. Bars with different letters indicate *P* < 0.05 (n = 6).

**Figure 3:**
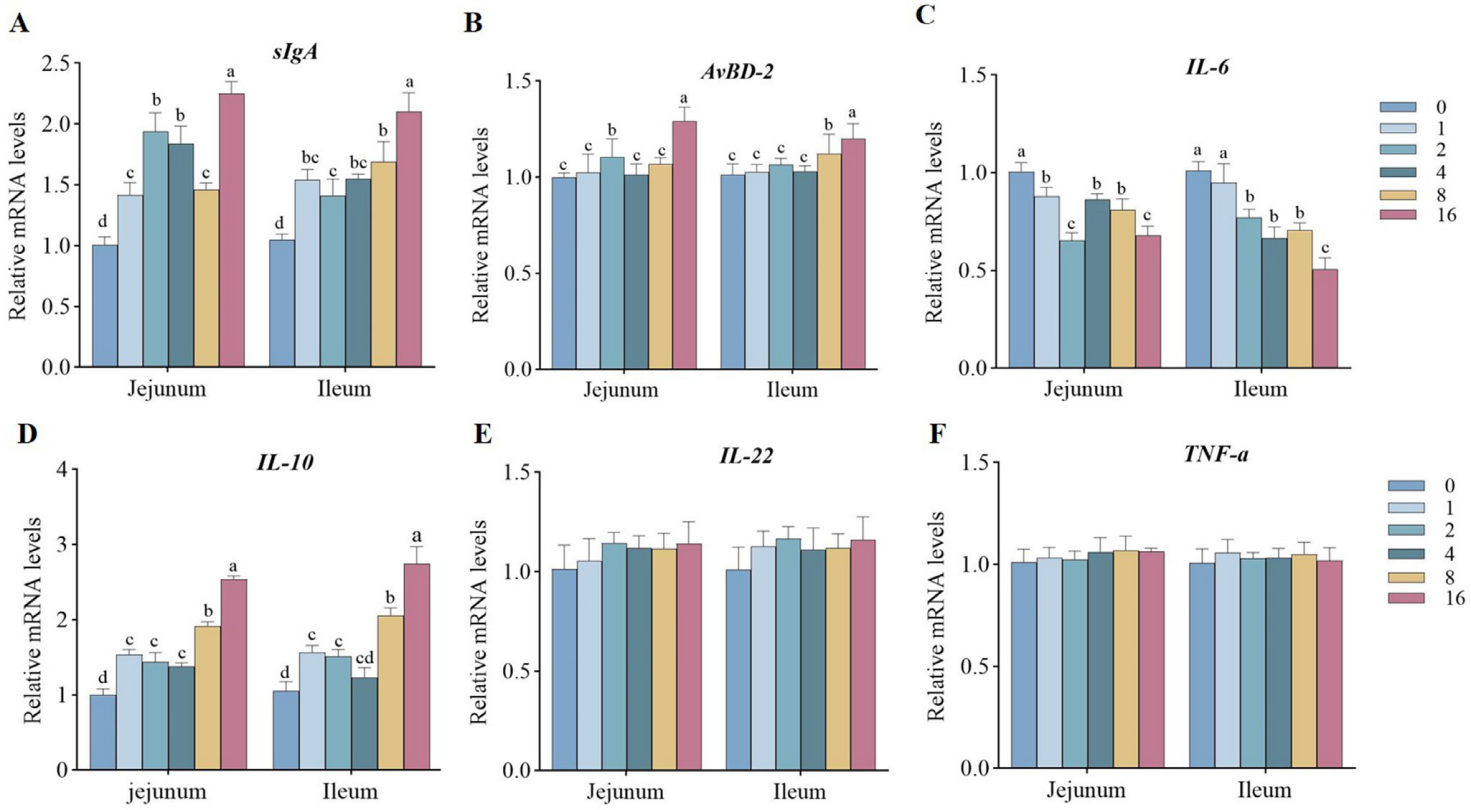
Expression of mRNA levels involved in intestinal immune barrier function of ducks fed differing levels of EDDI diets. Abbreviations: AvBD-2, avian β-defensin 2; IL-6, interleukin-6; IL-10, interleukin-10; IL-22, interleukin-22; sIgA, secretory immunoglobulin A; TNF-a, tumor necrosis factor a. Bars with different letters indicate *P* < 0.05 (n = 6).

The influence of EDDI on intestinal physical barrier function of ducks was presented in Figure 2. Compared with the control group, the relative expression levels of *ZO-1, ZO-2,* and *ZO-3* in intestine were markedly increased by the addition of 4, 8, and 16 mg/kg EDDI (*P* < 0.05). And the addition of EDDI observably also decreased the relative expression level of *OCLN* in intestine (*P* < 0.05). Compared with the control group, the relative expression of *MUC2* in intestine was significantly increased by the addition of EDDI (*P* < 0.05) (Figure 2). However, no differences in the relative expression level of *TFF2* were observed among the treatment groups (*P* > 0.05). The influence of EDDI on intestinal immune barrier of ducks was presented in Figure 3. Compared with the control group, the addition of EDDI observably increased the relative expression of *sIgA* and *IL-10* in intestine (*P* < 0.05). And 16 mg/kg EDDI markably increased the relative expression of *AvBD-2* in intestine compared with the control group (*P* < 0.05). The addition of 2, 4, 8 and 16 mg/kg EDDI also markedly decreased the relative expression of *IL-6* in intestine (*P* < 0.05).

### The Concentration of Short-Chain Fatty Acids in Cecum Response to Dietary EDDI Supplementation

**Figure 4:**
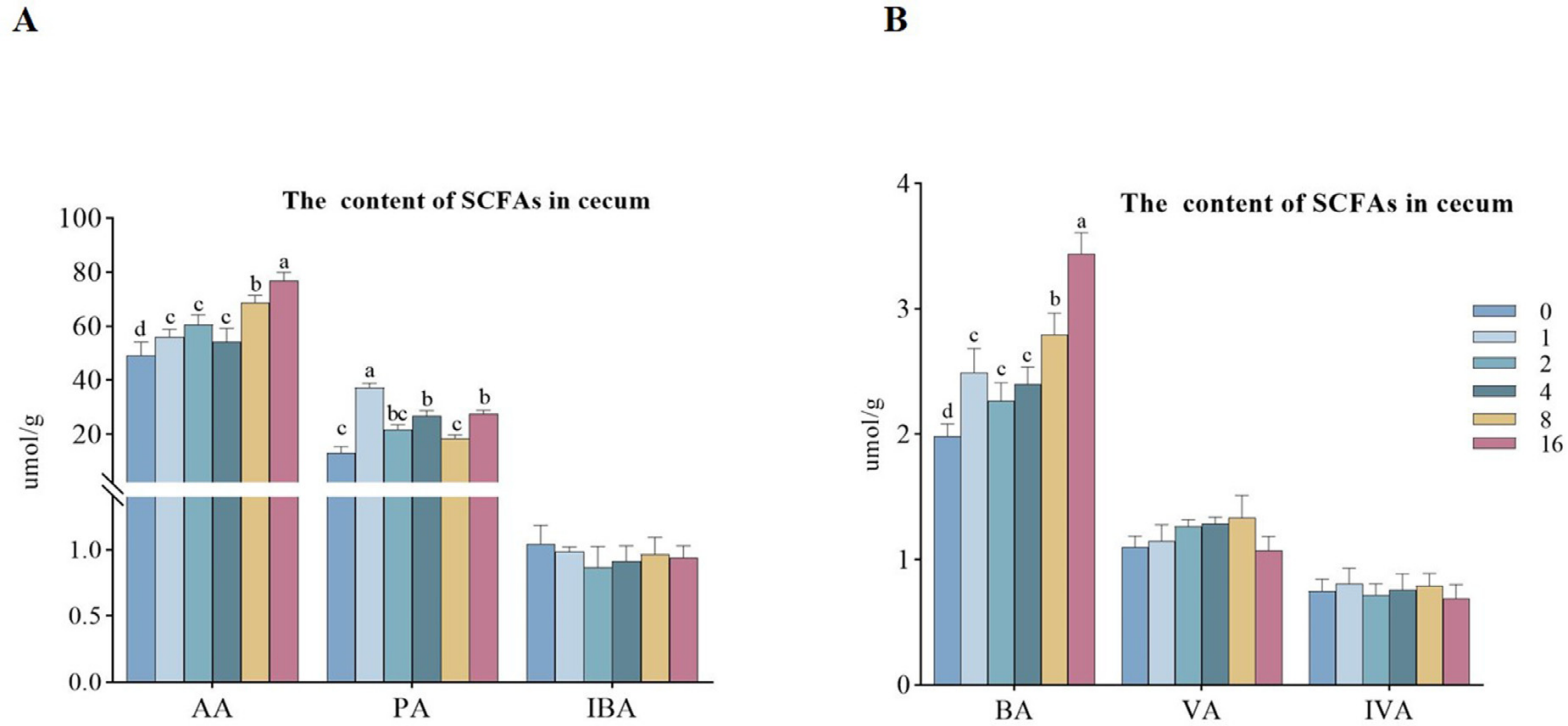
Quantitative analysis of short-chain fatty acids (SCFAs) in the cecum of ducks fed differing levels of EDDI diets. Abbreviations: Aa, acetic acid; Ba, butyric acid; IBa, isobutyric acid; IVa, isovaleric acid; Pa, propionic acid; Va, valeric acid. Bars with different letters indicate *P* < 0.05 (n = 6).

Figure 4 showed the effect of EDDI on the concentration of short-chain fatty acids (SCFAs) in cecum of ducks, compared with the control group, the addition of 1, 4, and 16 mg/kg EDDI observably increased (*P* < 0.05) the level of AA, PA, and BA in cecum of ducks. However, no differences in the levels of IBA, VA and IVA were observed among the treatment groups (*P* > 0.05).

### Sequencing, Microbial Abundance, and Diversity

**Figure 5:**
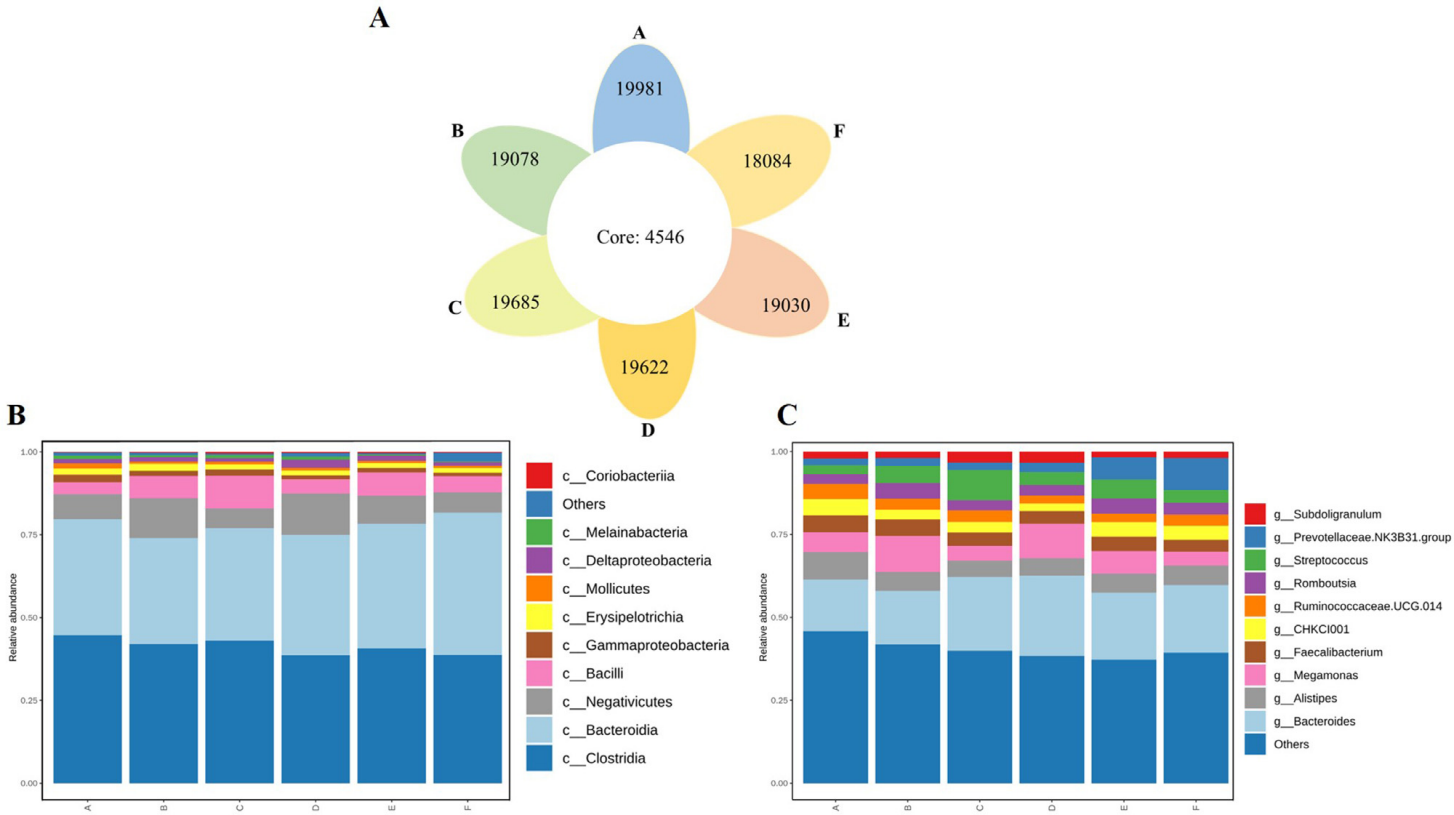
Common and specific OUT distribution of the fecal microbiota among 7 groups. (A) Venn Graph representation of the shared and exclusive OTU at the 97% similarity level of cecal microbiota; (B) The class level in each group; (C) The genus level in each group. Capital letters A–F refer to 0, 1, 2, 4, 8, and 16 mg/kg EDDI groups, respectively.

After 2,214,394 raw reads were spliced, quality controlled and de-chimerized, 2,182,697 joined tags were obtained, with an average of 60,630 joined tags per sample (Table S3). OTU cluster analysis showed that there were 115,480 unique OTU candidates with 97% sequence similarity, among which 4,546 candidates were shared by 6 treatment groups (Figure 5A). Based on the results of OTU annotation, Figure 5B and Figure 5C showed the top 10 class and genus of relative richness, respectively. And the most abundant class and genus of ducks were *Clostridia* and *Bacteroidetes*, respectively.

### Intestinal Microbial Barrier Function Response to Dietary EDDI Supplementation

Table 5 and Figure S1A showed the addition of EDDI observably up-regulated the richness of *Firmicutes, Actinobacteria,* and *Verrucomicrobia*, and the relative richness of *Deferribactere* down-regulated (*P* < 0.05) compared with the control group at the phylum level. Additionally, the order-taxa level of *Clostridiales, Selenomonadales,* and *Lactobacillales* were up-regulated (*P* < 0.05), and the relative richness of *Bacteroidales, Betaproteobacteriales, Mollicutes RF39,* and *Gastranaerophilales* were down-regulated in the EDDI treatment groups compared with the control group (*P* < 0.05) (Figure S1B and Table S4).

**Table 5:** The aligned percentages that annotated at phylum level (%).

Phylum	EDDI (mg/kg)						SEM	*P*-value
	0	1	2	4	8	16		
Firmicutes	57.78^b^	62.84^a^	60.42^a^	56.85^ab^	57.62^ab^	54.69^b^	0.910	0.021
Bacteroidetes	35.01	32.05	33.96	36.35	37.69	40.99	1.130	0.121
Proteobacteria	3.25	2.96	2.99	2.97	2.88	2.62	0.130	0.413
Tenericutes	1.02	0.70	0.78	0.83	0.66	0.70	0.080	0.717
Cyanobacteria	0.47	0.58	0.92	0.93	0.36	0.23	0.001	0.120
Actinobacteria	0.22^bc^	0.40^b^	0.69^ab^	0.52^ab^	0.56^ab^	1.00^a^	0.006	0.015
Epsilonbacteraeot	0.10^b^	0.13^b^	0.14^b^	0.06^b^	0.54^a^	0.06^b^	0.003	0.034
Verrucomicrobia	0.039^b^	0.044^b^	0.023^b^	0.024^b^	0.058^b^	0.344^a^	0.001	0.019
Deferribactere	0.36^a^	0.078^b^	0.035^bc^	0.112^ab^	0.051^b^	0.026^c^	<0.001	0.022
Acidobacteria	0.009	0.029	0.001	0.030	0.003	0.025	0.002	0.311

a-c Mean values (n = 6) in a row without a common superscript are significantly different in variance analysis (*P* ˂ 0.05).

### Correlations Between Microbiota With the Characteristic Indicators of Cherry Valley Ducks

**Figure 6:**
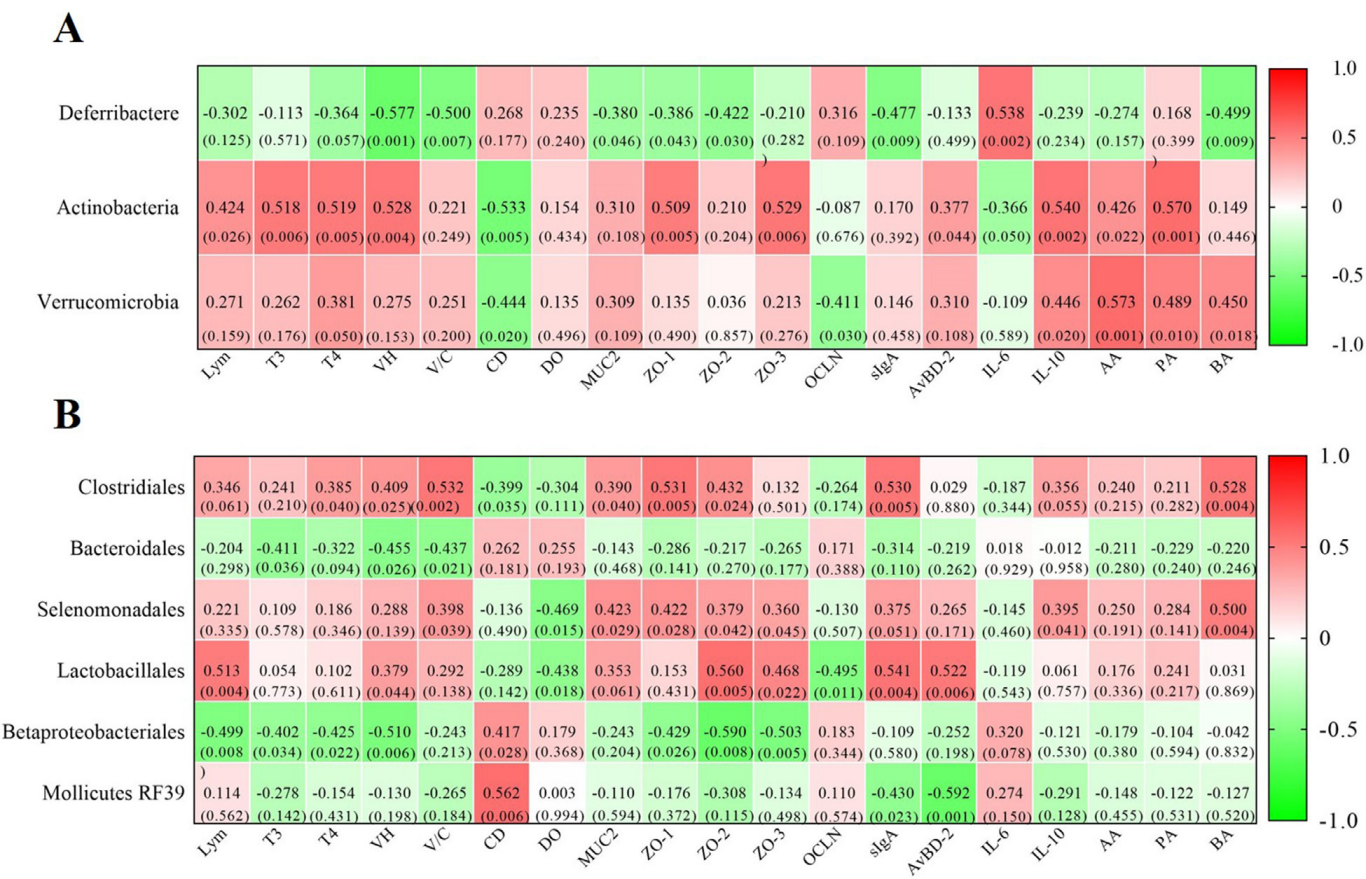
Correlations between cecal microbiota with the serum and intestinal related indicators: (A) At the phylum level. (B) At the order level. Each cell contains the corresponding correlation and *P*-value. The table is color-coded by correlation according to the color legend. Abbreviations: Aa, acetic acid; AvBD-2, avian β-defensin 2; Ba, butyric acid; CD, crypt depth; DO, diamine oxidase; IL-6, interleukin-6; IL-10, interleukin-10; Lym, lymphocytes; MUC2, mucin2; OCLN, occluding; Pa, propionic acid; sIgA, secretory immunoglobulin A; T3, triiodothyronine; T4, thyroxine; VH, villus height; V/C, villus height-to-crypt depth ratio; ZO-1, zonula occludens 1; ZO-2, zonula occludens 2; ZO-3, zonula occludens 3.

We used Pearson correlation tests to identify intestinal microbiota significantly associated with the phenotypic characteristics of ducks. At the phylum level, *Verrucomicrobia* richness was positively related (*P* < 0.05) with the concentrations of T_4_, *IL-10*, AA, PA and BA, but adversely related with CD value (*P* = 0.020) and *Ocln* expression (*P* = 0.030). The level of Lym, T_3_, T_4_, *Zo-1, Zo-3, AvBD-2, IL-10*, AA, and PA showed observably positive correlations (*P* < 0.05) with *Actinobacteria* richness, as well as VH value (*P* < 0.05), but related adversely with CD value and *IL-6* levels (*P* < 0.05). *Deferribactere* abundance related positively (*P* < 0.01) with the expression of *IL-6*, and related adversely (*P* < 0.01) with the VH and V/C value, the level of *sIgA* and Ba (Figure 6A). At the order level (Figure 6B), *Clostridiales* richness was positively related (*P* < 0.05) with T_4_, *Muc2, Zo-1, Zo-2, sIgA* and Ba levels, VH, and V/C values, but adversely related (*P* < 0.05) with the CD value. The T_3_ level, VH, and V/C values displayed markedly adverse correlations (*P* < 0.05) with *Bacteroidales* richness. The *Muc2, Zo-1, Zo-2, Zo-3, IL-10,* and Ba levels, V / C values were positively related (*P* < 0.05) with *Selenomonadales* richness, but adversely related with the level of DO (*P* < 0.05). *Lactobacillales* richness was positively related (*P* < 0.05) with Lym, VH, *Zo-2, Zo-3, sIgA,* and *AvBD-2*, but adversely related (*P* < 0.05) with DO and *Ocln*. The Lym, T_3_, T_4_, *Zo-1, Zo-2,* and *Zo-3* levels, VH value were adversely related (*P* < 0.05) with *Betaproteobacteriales* richness, but positively related with CD value (*P* < 0.05). Additionally, *Mollicutes RF39* richness related positively (*P* < 0.05) with the CD value, but related adversely (*P* < 0.05) with the expression of *sIgA* and *AvBD-2*.

## DISCUSSION

Our results demonstrated that EDDI diets promoted growth performance in ducks. Behroozlak et al. (2020) found that the diet supplemented with 5 mg/kg potassium iodide had no effects on growth performance of broiler. Similar results were reported that dietary 2.96 mg/kg calcium iodate had no significant effects on growth performance of broiler chickens (Eila et al., 2012). Above results seems to be inconsistent with our research findings. The possible reason was that EDDI was an organic chelating iodine, which has the characteristics of small molecular weight and high absorption, thus playing a positive role in the growth performance of ducks.

As an important part of thyroid hormone synthesis, iodine plays an important role in the growth and metabolism of animals. Usually, iodine exists in blood in the form of T3 and T4, with the proportion of T3 being low and the proportion of T4 being above 95% (Lin et al., 2014). The current experimental results showed that dietary EDDI supplementation significantly increased blood T3 and T4 concentrations. It is suggested that adding EDDI to the diet can ensure the iodine intake of meat ducks, promote the full synthesis of thyroid hormones in meat ducks and improve thyroid function, which on the one hand can promote the metabolism of sugar, fat and protein, thus promoting the digestion and absorption of nutrients (Eila et al., 2012). On the other hand, it can promote tissue differentiation, growth and maturity, and play an allowable role in growth hormone, so as to act on growth hormone secondarily and improve the growth and development of meat ducks (Zimmermann, 2011). Additionally, the number of lymphocytes and basophils were significantly increased, suggested that EDDI had a beneficial effect on the immune function of ducks. In consistency with the present study, Ali et al. (2017) reported that adding 2 mg/kg potassium iodide supplementation significantly increased the number of lymphocytes and heterophiles in ducks, thereby enhancing immune function.

As we all know, the main place for digestion of nutrients is intestine, so the health of intestinal environment is very important for the metabolism of nutrients, immune response and the regulation of body homeostasis (Kim and Ho, 2010). However, intestinal health mainly depends on intestinal development, intestinal permeability and barrier function (Liu et al., 2020). Intestinal integrity is mainly reflected by indicators such as VH, CD, and V/C. Generally speaking, lower VH and V/C values and higher CD value indicate worse intestinal development integrity and worse intestinal digestion and nutrition absorption capacity (Chang et al., 2022). In this study, dietary supplementation of EDDI exerted beneficial effects on intestinal morphology in VH and V/C in intestine, which suggested that EDDI might promote the digestion of nutrients by improving the integrity of intestine. Serum D-LA and DAO levels reflect intestinal permeability (Odenwald and Turner, 2017). In our study, the addition of EDDI was found to improve the intestinal permeability of ducks by decreasing DO and D-LA level, suggesting that EDDI may change the intestinal structure by improving the intestinal permeability.

Intestinal barrier is also very important to maintain intestinal health, including mechanical, chemical, immunological and microbial barriers (Ghosh et al., 2021; Ma et al., 2022). Among them, intestinal mechanical barrier depend on the expression of tight junction protein (Camara-Lemarroy et al., 2018). The chemical barrier mainly works through enzymes and mucus in the intestine (Birchenough et al., 2015). Immune barrier is mainly played by immune active molecules such as immunoglobulin, complement and cytokines (Uematsu and Fujimoto, 2010). Microbial barrier mainly means that the normal flora living in the intestinal tract secretes some bactericidal and bacteriostatic substances, which act as a barrier to pathogenic bacteria (Wang et al., 2018). In this study, EDDI enhanced mechanical barrier (*ZO-1, ZO-2, ZO-3,* and *OCLN*), chemical barrier (*MUC2*) and immune barrier (*sIgA, AvBD-2, IL-10,* and *IL-6*). Additionally, EDDI also improved microbial barrier function by changing the alpha and beta diversity, up-regulating the abundance of *Firmicutes, Actinobacteria, Epsilonbacteraeot,* and *Verrucomicrobia*, down-regulating the abundance of *Deferribactere* in phylum level. And EDDI also changed the abundance of *Clostridiales, Selenomonadales, Lactobacillales, Bacteroidales, Betaproteobacteriales, Mollicutes RF39,* and *Gastranaerophilales* in order level. Interestingly, the addition of EDDI was also found to increase the content of AA, PA, and BA in cecum of ducks, which may be attributed to the increase of the abundance of bacteria producing AA, PA, and BA. *Actinobacteria* is absolute players in maintaining gut barrier homeostasis because they have an ability to produce SCFAs, especially Aa and Ba (Ashida et al., 2012; Barczynska et al., 2015). *Verrucomicrobia* is mainly the *Akkermansia muciniphila* of the *Verrucomicrobiaceae* that produces AA,

PA and BA. Importantly, *A. muciniphila* regulated metabolism and immune function, as well as protected intestinal health (Zhai, 2019). *Deferribactere* is an opportunistic pathogen, which can promote inflammation (Long et al., 2010). *Clostridiales* is also a bacterium that mainly produces butyrate, and it has been reported that the decrease of butyrate will destroy the intestinal integrity, resulting in the increase of colon permeability (Pichler et al., 2020). *Lactobacillus* produces lactic acid, prevents the invasion and colonization of pathogenic bacteria to the intestinal tract, and maintains the micro-ecological balance of the intestines (Castro-Bravo et al., 2018). It has been reported that intestinal microorganisms not only influenced the barrier function of the host intestine, but also regulated intestinal immune response, and then affected other physiological characteristics (Maloy and Powrie, 2011). This study discovered that the addition of EDDI had a significant impact on the abundance of microbiota, and these microbiota were positively or negatively correlated with the related characteristics of duck's thyroid function, immune function, intestinal development, intestinal permeability and barrier function. This indicated that there was a complex and beneficial interaction between intestinal microorganisms and animal physiological networks to enhance the intestinal health and promote the growth and development of ducks.

## CONCLUSIONS

This study revealed that the addition of EDDI to the diet had a strong positive effect on the intestinal health of cherry valley ducks. That is, the addition of 7.40 to 9.60 mg/kg iodine (EDDI form) improved the growth and development of the meat ducks by enhancing the thyroid function and immune function, improving the integrity and permeability of the intestinal structure, promoting the digestion and absorption of nutrients, changing the expression levels of genes related to the intestinal physical, chemical and immune barriers, and increasing the abundance of SCFA-producing microorganisms and reducing the abundance of pathogenic bacteria.

## Appendix Supplementary materials

Image, application 1

## Footnotes

Supplementary material associated with this article can be found in the online version at doi:10.1016/j.psj.2023.103022.

## References

[bib0001] Ali W., Ali K., Hekal A., Easa F., EL-AIK M., Ali R. (2017). Effect of dietary iodine supplementation on productive performance of Pekin and Domyati ducks during growth period. J. Anim. Poult. Prod..

[bib0002] Ammerman C., Baker D., Lewis A. (1995).

[bib0003] Ammerman C., Miller S.M. (1972). Biological availability of minor mineral ions: a review. J. Anim. Sci..

[bib0004] Ashida H., Ogawa M., Kim M., Mimuro H., Sasakawa C. (2012). Bacteria and host interactions in the gut epithelial barrier. Nat. Chem. Biol..

[bib0005] Bao Y., Choct M. (2009). Trace mineral nutrition for broiler chickens and prospects of application of organically complexed trace minerals: a review. Anim. Prod. Sci..

[bib0006] Barczynska R., Slizewska K., Litwin M., Szalecki M., Zarski A., Kapusniak J. (2015). The effect of dietary fibre preparations from potato starch on the growth and activity of bacterial strains belonging to the phyla Firmicutes, Bacteroidetes, and Actinobacteria. J. Funct. Foods.

[bib0007] Behroozlak M., Daneshyar M., Farhomand P. (2020). The effects of dietary iodine and its consumption duration on performance, carcass characteristics, meat iodine, thyroid hormones and some blood indices in broiler chickens. J. Anim. Physiol. Anim. Nutr..

[bib0008] Birchenough G., Johansson M.E., Gustafsson J., Bergström J., Hansson G. (2015). New developments in goblet cell mucus secretion and function. Mucosal Immunol..

[bib0009] Camara-Lemarroy C., Metz L., Meddings J., Sharkey K., Wee Yong V. (2018). The intestinal barrier in multiple sclerosis: implications for pathophysiology and therapeutics. Brain.

[bib0010] Castro-Bravo N., Wells J., Margolles A., Ruas-Madiedo P. (2018). Interactions of surface exopolysaccharides from Bifidobacterium and Lactobacillus within the intestinal environment. Front. Microbiol..

[bib0012] Chang L., Ding Y., Wang Y., Song Z., Li F., He X., Zhang H. (2022). Effects of different oligosaccharides on growth performance and intestinal function in broilers. Front. Vet. Sci..

[bib0013] Eila N., Asadi H., Shivazad M., Zarei A., Akbari N. (2012). Effect of different calcium iodate levels on performance, carcass traits and concentration of thyroid hormones in broiler chickens. Ann. Biol. Res..

[bib0015] Fomichev Y., Bogolyubova N., Mishurov A., Rykov R.A. (2019). Bio correction of enzymatic and microbial processes in rumen and intermediary metabolism in sheep with the use of antioxidant and organic iodine dietary supplements. Russian Agric. Sci..

[bib0016] Ghosh S., Whitley C., Haribabu B., Jala V. (2021). Regulation of intestinal barrier function by microbial metabolites. Cell. Mol. Gastroenterol. Hepatol..

[bib0017] Goo D., Kim J., Choi H., Park G., Han G., Kil D. (2019). Effect of stocking density and sex on growth performance, meat quality, and intestinal barrier function in broiler chickens. Poult. Sci..

[bib0019] Iannaccone M., Ianni A., Elgendy R., Martino C., Giantin M., Cerretani L., Dacasto M., Martino G. (2019). Iodine supplemented diet positively affect immune response and dairy product quality in Fresian Cow. Animals.

[bib0020] Inal F., Coşkun B., Gülşen N., Kurtoğlu V. (2001). The effects of withdrawal of vitamin and trace mineral supplements from layer diets on egg yield and trace mineral composition. Br. Poult. Sci..

[bib0021] Kim Y.S., Ho S.B. (2010). Intestinal goblet cells and mucins in health and disease: recent insights and progress. Curr. Gastroenterol. Rep..

[bib0022] Lambert G. (2009). Stress-induced gastrointestinal barrier dysfunction and its inflammatory effects. J. Anim. Sci..

[bib0023] Ledesma C., Rosario C., Gracia-Mora J., Tapia G., Sumano H., Gutierrez L. (2018). Influence of chlorine, iodine, and citrate-based water sanitizers on the oral bioavailability of enrofloxacin in broiler chickens. J. Appl. Poult. Res..

[bib0024] Lin S., Wang C., Tan S., Liang Y., Yao H., Zhang Z., Xu S. (2014). Selenium deficiency inhibits the conversion of thyroidal thyroxine (T4) to triiodothyronine (T3) in chicken thyroids. Biol. Trace. Elem. Res..

[bib0025] Liu W.C., Guo Y., Zhao Z.H., Jha R., Balasubramanian B. (2020). Algae-derived polysaccharides promote growth performance by improving antioxidant capacity and intestinal barrier function in broiler chickens. Front. Vet. Sci..

[bib0026] Liu Y., Lin Q., Huang X., Huang X. (2021). Effects of dietary ferulic acid on the intestinal microbiota and the associated changes on the growth performance, serum cytokine profile, and intestinal morphology in ducks. Front. Microbiol..

[bib0027] Long Y., Xie L., Liu N., Yan X., Li M. (2010). Comparison of gut-associated and nest-associated microbial communities of a fungus-growing termite (*Odontotermes yunnanensis*). Insect. Sci..

[bib0028] Ma J., Piao X., Mahfuz S., Long S., Wang J. (2022). The interaction among gut microbes,the intestinal barrier and short chain fatty acids. Anim. Nutr..

[bib0029] Maloy K.J., Powrie F. (2011). Intestinal homeostasis and its breakdown in inflammatory bowel disease. Nature.

[bib0030] Odenwald M.A., Turner J.R. (2017). The intestinal epithelial barrier: a therapeutic target?. Nat. Rev. Gastro. Hepat..

[bib0031] Pichler M.J., Yamada C., Shuoker B., Alvarez-Silva C., Hachem M.A. (2020). Butyrate producing colonic Clostridiales metabolise human milk oligosaccharides and cross feed on mucin via conserved pathways. Nat. Commun..

[bib0032] Qin S., Zhang K., Applegate T.J., Ding X., Zeng Q. (2019). Dietary administration of resistant starch improved cecal barrier function by enhancing intestinal morphology and modulating microbiota composition in meat duck. Br. J. Nutr..

[bib0033] Sirakov M., Plateroti M. (2011). The thyroid hormones and their nuclear receptors in the gut: from developmental biology to cancer. Biochim. Biophys. Acta..

[bib0034] Uematsu S., Fujimoto K. (2010). The innate immune system in the intestine. Microbiol. Immun..

[bib0035] Wang J., Ji H., Hui S., Liu W., Zhang D. (2018). Probiotic Lactobacillus plantarum promotes intestinal barrier function by strengthening the epithelium and modulating gut microbiota. Front. Microbiol..

[bib0036] Yu H., Xie Y., Wu B., Zhao H., Chen X., Tian G., Liu G., Cai J., Jia G. (2022). Dietary supplementation of ferrous glycinate improves intestinal barrier function by modulating microbiota composition in Cherry Valley ducks. Anim. Nutr..

[bib0038] Zhai Q., Feng S., Arjan A., Chen W. (2019). A next generation probiotic, *Akkermansia muciniphila*. Crit. Rev.Food. Sci. Nutr..

[bib0039] Zimmermann M.B. (2011). The role of iodine in human growth and development. Semin. Cell. Dev. Biol..

