# Supplementary material for: Dietary ethylenediamine dihydroiodide improves intestinal health in Cherry Valley ducks
Source: Poult Sci. 2023 Aug 11;102(11):103022. doi: 10.1016/j.psj.2023.103022 (PMC10477681; doi:10.1016/j.psj.2023.103022)
Supplement: Supplementary file 1 [file mmc1.docx]

**Table S1 Ingredients and nutrient levels of basic diets (air-dry basis, %)**

| Ingredient | Content | | Nutrient levels^2^ | Content | |
| --- | --- | --- | --- | --- | --- |
|  | 1-14 d | 15-35 d |  | 1-14 d | 15-35 d |
| Corn, 7.8% CP | 60.84 | 66.58 | AME (MJ/kg) | 12.14 | 12.14 |
| Soybean meal, 43% CP | 34.85 | 27.93 | Crude protein (%) | 20.14 | 17.55 |
| Wheat bran | 0.64 | 0.64 | Ca (%) | 0.90 | 0.85 |
| CaCO_3_ | 0.77 | 0.71 | Available P (%) | 0.42 | 0.40 |
| CaHPO_4_ (2H_2_O) | 2.04 | 2.04 | Lysine (%) | 1.10 | 0.91 |
| Sodium chloride | 0.30 | 0.30 | Methionine (%) | 0.46 | 0.41 |
| Choline chloride, 50% | 0.34 | 0.34 | Threonine (%) | 0.28 | 0.20 |
| Vitamin premix^1^ | 0.15 | 0.10 | Tryptophan (%) | 0.79 | 0.71 |
| Mineral premix^1^ | 0.03 | 0.03 | Iodine content (mg/kg)^3^ | 0.82 | 0.91 |
| *L*-lysine·HCl ^2^, 98.5% | 0.30 | 0.30 |  |  |  |
| *DL*-methionine, 99% | 0.22 | 0.13 |  |  |  |
| Total | 100.00 | 100.00 |  |  |  |

^1^ Provided the following per kilogram of the diet: vitamin A, 9000 IU; vitamin D_3_, 2000 IU; vitamin E, 10 mg; vitamin B_1_, 2 mg; vitamin B_2_, 4.8 mg; vitamin B_12_, 0.02 mg; pantothenic acid 20 mg; folic acid 1 mg; niacin 50 mg. The mineral premix provides the following per kg of diets: Cu (CuSO_4_) 8 mg; Fe (FeSO_4_) 60 mg; Mn (MnSO_4_) 60 mg; Zn（ZnSO_4_·7H_2_O）60 mg; Se (NaSeO_3_) 0.3 mg.

^2^ CP, iodine and energy were measured values, while the others were calculated values.

^3^ Total iodine from 1 to 14 days of age diets was 0.82, 1.90, 3.23, 4.35, 9.27, 17.16 mg/kg and from 15 to 35 days of age diets was 0.97, 2.05, 3.54, 5.47, 9.86, 17.65 mg/kg, respectively.

**Table S2 Information of primers for target genes**

| Genes | Accession no. | Primer sequences (5’ to 3’) | | Product size, bp |
| --- | --- | --- | --- | --- |
| *MUC2* | XM_005024513.2 | Forward: | GGGCGCTCAATTCAACATAAGTA | 150 |
|  |  | Reverse: | TAAACTGATGGCTTCTTATGCGG |  |
| *TFF2* | XM_005030468.2 | Forward: | CTGTCATCCTTGTTGTAGCCCTCA | 113 |
|  |  | Reverse: | GATTCCTGGGTGACCACAGTTCTT |  |
| *AvBD2* | AY641439.1 | Forward: | CCAGGTTTCTCCAGGATTGT | 117 |
|  |  | Reverse: | AACCCAAAGCAACTTCCAAC |  |
| sIgA | U27222.1 | Forward: | TCGCTCAAGGAACCCATCGT | 174 |
|  |  | Reverse: | GCGGGACCACGAGAACTTCA |  |
| *IL-6* | XM_013100522.2 | Forward: | CTGCGAGAACAGCATGGAGA | 191 |
|  |  | Reverse: | GAAAGGTGAAAAGCCCGCTG |  |
| *IL-10* | AJ621614.1 | Forward: | CCTGCCTGCCCACTGCTTGG | 92 |
|  |  | Reverse: | TGAGGGTGAAGTTTGAGGAAAT |  |
| *IL-22* | NM_001389540.2 | Forward: | TTCCCCTTATACGCCGCTGA | 259 |
|  |  | Reverse: | TTGGGTTAAAACACTGCCAGAT |  |
| *TNF-a* | XM_005019359.2 | Forward: | ACAGGACAGCCTATGCCAAC | 165 |
|  |  | Reverse: | ACAGGAAGGGCAACACATCT |  |
| *ZO-1* | XM 013104936.1 | Forward: | ACGCTGGTGAAATCAAGGAAGAA | 255 |
|  |  | Reverse: | AGGGACATTCAACAGCGTGGC |  |
| *ZO-2* | XM 013093747.1 | Forward: | ACAGTGAAAGAAGCTGGCGTAG | 131 |
|  |  | Reverse: | GCTGTATTCCCTGCTACGGTC |  |
| *ZO-3* | XM_013109403.1 | Forward: | CAACATCCCTGACATGGAAGACAT | 187 |
|  |  | Reverse: | TGTGTTCGTGTTGGTTGCGG |  |
| *OCLN* | XM 013109403.1 | Forward: | GGCTTCCTCATCGTCCTCTTG | 160 |
|  |  | Reverse: | TCTCGTACTGCGACTCGTCCA |  |
| *β-actin* | EF667345.1 | Forward: | AGAAATTGTGCGTGACATCAA | 227 |
|  |  | Reverse: | GGACTCCATACCCAAGAAAGAT |  |

*MUC2* = mucin2; *TFF2* = trefoil factor 2; *AvBD2* = avian β-defensin 2; *sIgA* = secretory immunoglobulin A; *IL-6* = interleukin-6; *IL-10* = interleukin-10; *IL-22* = interleukin-22; *TNF-a* = tumor necrosis factor *a*; *ZO-1* = zonula occludens 1; *ZO-2* = zonula occludens 2; *ZO-3* = zonula occludens 3; *OCLN* = occluding.

**Table S3 Quality control and preprocessing of metagenomic datasets**

| Sample_name | Raw_reads | Clean_Reads | Base (M) | Q 20, % | GC, % | Effective, % |
| --- | --- | --- | --- | --- | --- | --- |
| A1 | 60,913 | 59,788 | 21,701,664 | 95.45 | 52.21 | 85.85 |
| A2 | 62,854 | 62,347 | 23,859,555 | 95.58 | 51.72 | 91.22 |
| A3 | 51,589 | 50,842 | 19,203,208 | 94.91 | 52.11 | 89.26 |
| A4 | 63,953 | 63,394 | 24,079,010 | 95.74 | 52.25 | 90.74 |
| A5 | 66,003 | 65,383 | 24,949,555 | 95.6 | 52.15 | 90.67 |
| A6 | 60,422 | 59,754 | 22,675,160 | 95.28 | 51.33 | 90.03 |
| B1 | 61,368 | 60,825 | 23,020,776 | 95.52 | 51.96 | 90.03 |
| B2 | 65,767 | 65,253 | 24,868,594 | 95.93 | 52.11 | 91.19 |
| B3 | 64,514 | 63,838 | 24,264,158 | 95.72 | 51.63 | 90.43 |
| B4 | 65,245 | 64,262 | 24,561,199 | 95.38 | 52.06 | 90.16 |
| B5 | 66,575 | 65,863 | 25,713,959 | 95.36 | 52.29 | 92.02 |
| B6 | 79,539 | 78,845 | 26,015,346 | 96.56 | 52.31 | 78.53 |
| C1 | 58,758 | 57,583 | 21,421,098 | 95.16 | 51.83 | 87.56 |
| C2 | 53,063 | 51,787 | 19,457,345 | 95.07 | 51.19 | 87.9 |
| C3 | 64,130 | 63,414 | 23,406,740 | 95.65 | 52.34 | 88.4 |
| C4 | 62,021 | 60,974 | 22,685,224 | 95.36 | 52.15 | 87.75 |
| C5 | 53,786 | 53,221 | 19,889,456 | 95.69 | 52.1 | 89.07 |
| C6 | 60,118 | 59,185 | 23,134,399 | 95.1 | 51.6 | 91.49 |
| D1 | 62,987 | 61,404 | 22,481,421 | 95.67 | 52.44 | 85.95 |
| D2 | 59,089 | 58,432 | 22,882,728 | 95.4 | 52.34 | 92.76 |
| D3 | 62,950 | 62,256 | 24,134,477 | 95.36 | 51.86 | 91.57 |
| D4 | 61,509 | 60,976 | 23,453,778 | 95.53 | 51.84 | 91.63 |
| D5 | 68,714 | 66,836 | 25,351,155 | 95.44 | 51.98 | 88.15 |
| D6 | 57,755 | 56,525 | 21,264,408 | 95.26 | 51.29 | 88.09 |
| E1 | 50,732 | 50,174 | 19,298,567 | 95.01 | 52.38 | 91.13 |
| E2 | 64,101 | 63,473 | 23,942,595 | 95.75 | 52.19 | 89.81 |
| E3 | 52,757 | 51,708 | 18,901,118 | 95.15 | 52.25 | 85.95 |
| E4 | 64,147 | 63,211 | 23,642,409 | 95.64 | 52.72 | 88.42 |
| E5 | 67,578 | 66,213 | 25,104,170 | 95.61 | 52.42 | 88.87 |
| E6 | 60,938 | 59,436 | 22,248,733 | 95.41 | 52.04 | 87.41 |
| F2 | 64,120 | 63,063 | 23,434,261 | 95.8 | 52.8 | 87.43 |
| F3 | 58,637 | 58,066 | 21,650,843 | 95.87 | 52.36 | 89.15 |
| F4 | 60,938 | 59,841 | 22,200,055 | 95.56 | 51.73 | 87.53 |
| F5 | 62,467 | 61,736 | 23,677,234 | 95.7 | 52.67 | 90.91 |
| F6 | 53,096 | 52,523 | 20,006,258 | 95.32 | 51.61 | 90.21 |
| F7 | 61,261 | 60,266 | 23,005,941 | 95.5 | 52.01 | 89.72 |

Raw_reads = original sequencing data; Clean_Reads = final valid reads; Base, the total base number of effective tags; Q 20, the percentage of effective tags with the Q value of each base ≥ 20 (sequencing error rate ≤ 1%); Effective, the percentage of effective tags in original sequencing reads. A = 0 mg/kg EDDI group; B = 1 mg/kg EDDI group; C = 2 mg/kg EDDI group; D = 4 mg/kg EDDI group; E = 8mg/kg EDDI group; F = 16 mg/kg EDDI group.

**Table S4 The aligned percentages that annotated at order level（%）**

| Order | EDDI (mg/kg) | | | | | | SEM | *P*-value |
| --- | --- | --- | --- | --- | --- | --- | --- | --- |
|  | 0 | 1 | 2 | 4 | 8 | 16 |  |  |
| Clostridiales | 38.71^bc^ | 42.02 ^ab^ | 43.04^ab^ | 41.65^b^ | 40.96^b^ | 44.65^a^ | 0.596 | 0.017 |
| Bacteroidales | 42.95^a^ | 39.92^ab^ | 33.96^bc^ | 36.31^b^ | 37.69^b^ | 31.99^c^ | 0.621 | <0.001 |
| Selenomonadales | 6.08^bc^ | 7.56^b^ | 5.92^c^ | 11.99^ab^ | 8.41^b^ | 12.45^a^ | 0.213 | 0.025 |
| Lactobacillales | 3.45^c^ | 5.70^bc^ | 9.77^a^ | 4.20^bc^ | 6.98^b^ | 6.99^b^ | 0.142 | 0.016 |
| Erysipelotrichales | 1.90 | 1.03 | 1.56 | 1.03 | 1.49 | 1.44 | 0.003 | 0.654 |
| Desulfovibrionales | 1.38 | 1.03 | 1.05 | 1.44 | 1.54 | 1.04 | 0.001 | 0.352 |
| Betaproteobacteriales | 2.14^a^ | 1.21^b^ | 1.70^ab^ | 1.05 ^b^ | 0.94 ^b^ | 0.89^b^ | 0.001 | 0.019 |
| Mollicutes RF39 | 1.50^a^ | 0.70 ^b^ | 0.68 ^b^ | 0.18^c^ | 0.66 ^b^ | 0.70 ^b^ | 0.004 | 0.034 |
| Gastranaerophilales | 1.00 ^a^ | 0.59^ab^ | 0.92 ^a^ | 0.93 ^a^ | 0.37 ^b^ | 0.23 ^b^ | 0.001 | 0.027 |
| Coriobacteriales | 0.16 | 0.20 | 0.50 | 0.33 | 0.45 | 0.21 | 0.005 | 0.215 |

Note: ^a-c^ Mean values (*n =* 6) in a row without a common superscript are significantly different in variance analysis (*P* ˂ 0.05).


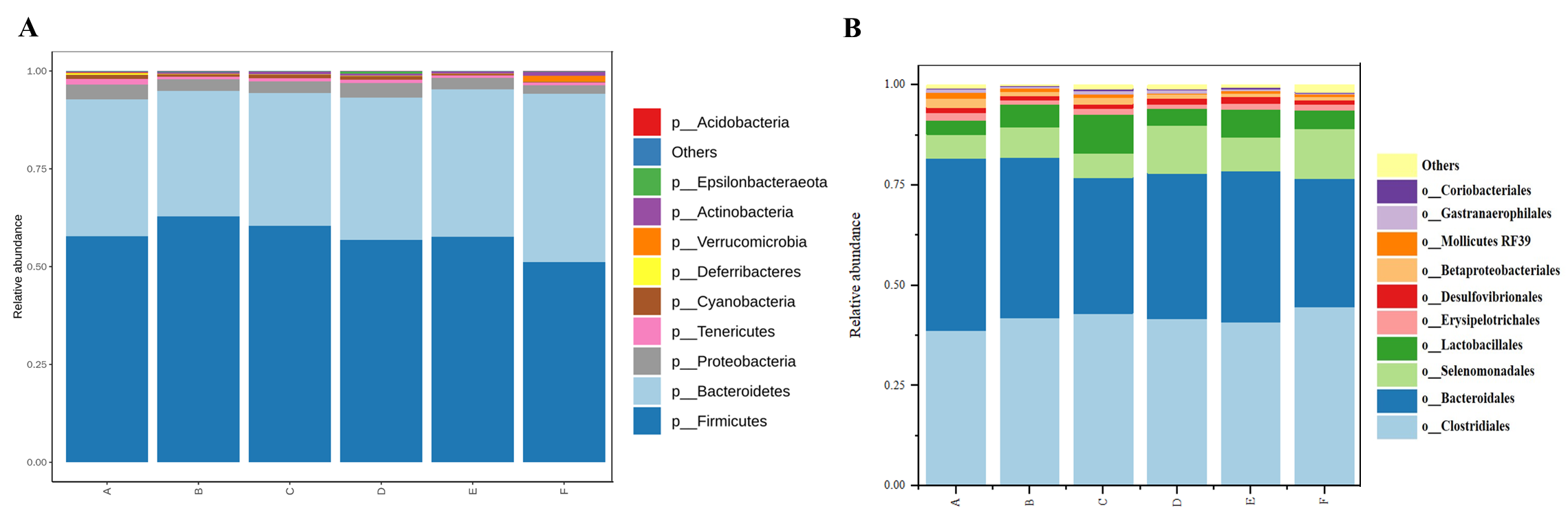


**Fig. S1**. Community bar-plot analysis shows relative abundance of cecal microbiota in phylum and order level: (A) The phylum level in each group. (B) The order level in each sample. Capital letters A–F refer to 0, 1, 2, 4, 8 and 16 mg/kg EDDI groups, respectively.
